# RIG-I-Like Receptor-Mediated Recognition of Viral Genomic RNA of Severe Acute Respiratory Syndrome Coronavirus-2 and Viral Escape From the Host Innate Immune Responses

**DOI:** 10.3389/fimmu.2021.700926

**Published:** 2021-06-25

**Authors:** Takahisa Kouwaki, Tasuku Nishimura, Guanming Wang, Hiroyuki Oshiumi

**Affiliations:** Department of Immunology, Graduate School of Medical Sciences, Faculty of Life Sciences, Kumamoto University, Kumamoto, Japan

**Keywords:** Innate immunity, Type I interferon, RIG-I, MDA5, SARS-CoV-2, coronavirus

## Abstract

RIG-I-like receptors (RLR), RIG-I and MDA5, are cytoplasmic viral RNA sensors that recognize viral double-stranded RNAs and trigger signals to induce antiviral responses, including type I interferon production. Severe acute respiratory syndrome coronavirus-2 (SARS-CoV-2) caused the coronavirus disease 2019 pandemic. However, the RLR role in innate immune response to SARS-CoV-2 has not been fully elucidated. Here, we studied the roles of RLR in cytokine expression responding to SARS-CoV-2 and found that not only MDA5 but also RIG-I are involved in innate immune responses in some types of human cells. Transfection of total RNAs extracted from SARS-CoV-2-infected cells into epithelial cells induced IFN-β, IP-10, and Ccl5 mRNA expression. The cytokine expression was reduced by knockout of either RIG-I or MDA5, suggesting that both proteins are required for appropriate innate immune response to SARS-CoV-2. Two viral genomic RNA regions strongly induced type I IFN expression, and a 200-base fragment of viral RNA preferentially induced type I IFN in a RIG-I-dependent manner. In contrast, SARS-CoV-2 infectious particles hardly induced cytokine expression, suggesting viral escape from the host response. Viral 9b protein inhibited RIG-I and MAVS interaction, and viral 7a protein destabilized the TBK1 protein, leading to attenuated IRF-3 phosphorylation required for type I IFN expression. Our data elucidated the mechanism underlying RLR-mediated response to SARS-CoV-2 infection and viral escape from the host innate immune response.

## Introduction

RIG-I and MDA5 are cytoplasmic viral RNA sensors that induce type I interferon (IFN) and other pro-inflammatory cytokine expression (1). RIG-I recognizes 5′ tri- or diphosphate double-stranded RNA (dsRNA) and prefers relatively short dsRNA (less than 1 kbp), whereas MDA5 binds longer dsRNA (greater than 1 kbp) and makes nucleoprotein filaments (2, 3). Host and viral RNA fragments cleaved by RNase L, such as dsRNA with 3′ phosphate, are also recognized by RIG-I (4). Previous studies showed that single-stranded RNA with secondary structures forming dsRNA regions, such as the 3’ UTR region of hepatitis C virus (HCV) and the ϵ region of hepatitis B virus genomic RNA, is preferentially recognized by RIG-I (5, 6).

The RIG-I and MDA5 proteins comprise N-terminal caspase-activation and recruitment domains (CARDs), RNA helicase, and a C-terminal domain (CTD) (7, 8). The RNA helicase and CTDs bind to viral RNA (9), and after the recognition of viral RNA, RIG-I requires post-translational modification (10). K63-linked polyubiquitination at N-terminal CARDs is essential to induce RIG-I-mediated type I IFN expression (11), and Riplet and TRIM25 ubiquitin ligases are reported to be involved in RIG-I polyubiquitination (12, 13). Polyubiquitin chain stabilizes the assembly of RIG-I CARDs and promotes the 2CARD tetramer structure (14, 15). The structure acts as a core for MAVS adaptor assembly, thereby activating the TBK1 protein (16). TBK1 is a protein kinase that phosphorylates IRF-3, and phosphorylated IRF-3 is relocated into the nucleus, activating type I IFN gene transcription (17). MDA5 proteins are regulated by phosphorylation (18), and it also uses a MAVS adaptor and induces type I IFN expression like RIG-I (1).

Severe acute respiratory syndrome coronavirus 2 (SARS-CoV-2) causes coronavirus disease 2019 (COVID-19) (19). SARS-CoV-2 belongs to the genus *Betacoronavirus* and induces respiratory symptoms (20). Viral RNAs are detected in respiratory tracts and other tissues of patients with COVID-19, including the blood, kidney, and liver (21). The spike protein of SARS-CoV-2 binds a cell surface receptor, angiotensin-converting enzyme 2 (ACE2), thereby invading host cells (19). ACE2 is expressed in various cells and tissue types (22). In addition to humans, SARS-CoV-2 can infect macaques, ferrets, and hamsters, and human ACE2 transgenic mice are also susceptible to SARS-CoV-2 (23). Recent studies on animal models or human cells showed that SARS-CoV-2 weakly induces type I IFN expression; however, its underlying mechanism is not fully elucidated (24–26). Although the important role of MDA5 in the antiviral innate immune response to SARS-CoV-2 in lung epithelial cells has been reported (27, 28), the role of RIG-I remains elusive. This study investigated the roles of RIG-I and MDA5 in the innate immune response to SARS-CoV-2, and found that the both receptors play crucial roles in the type I IFN expression after SARS-CoV-2 infection.

## Materials and Methods

### Cell Culture and Viruses

HEK293 cells were kindly provided by T. Seya (29) and maintained in D-MEM (low Glc) with 10% heat-inactivated fetal calf serum (FCS) and penicillin-streptomycin solution (P/S). HEK293FT and A549 cells were incubated in D-MEM (high Glc) with 10% FCS and P/S. HeLa cells were cultured in Eagle’s-MEM with 10% FCS and P/S. Vero cells and VeroE6/TMPRSS2 cells were cultured in D-MEM (low Glc) with 10% FCS and P/S. Influenza A virus and Sendai virus (SeV) was amplified using Vero cells, and the number of plaque-forming units was determined by plaque assays. SARS-CoV-2 JPN/TY/WK-521 strain was obtained from the National Institute of Infectious Diseases in Japan (30) and amplified with VeroE6/TMPRSS2 cells. Original virus stock was contaminated with mycoplasma (30), and thus mycoplasma was removed using MycoZap Mycoplasma Elimination Reagent (Lonza). We confirmed that mycoplasmas were eliminated from virus stock solution by PCR. SARS-CoV-2 viral titers were determined by a TCID50 assay, according to Behrens-Karber’s method. RIG-I, MDA5, and Riplet KO HEK293 cells were generated using the CRISPR-Cas9 system. The guide sequences for each KO are RIG-I KO: GTG ACT GCC TCG GTT GGT GTT, MDA5: GCG AAT TCC CGA GTC CAA CCA, Riplet KO: GCC CGC CTG TAT CTC GCG TG. The targeted regions within RIG-I, MDA5, and Riplet were sequenced, and the mutations were confirmed by DNA sequencing. We confirmed that mRNA, endogenous protein, or levels of both was barely detectable in each cell KO by RT-qPCR and Western blotting.

### SARS-CoV-2 Viral RNA Fragments

Total RNAs of VeroE6/TMPRSS2 cells infected with SARS-CoV-2 were isolated with TRIZOL, and cDNA was prepared using a random primer. cDNA fragments encoding the viral RNA segments were amplified with the primer described in **Supplemental Table S1**. The viral RNA segments were synthesized with T7 RNA polymerase, but the viral RNA segment (5001–6000) could not be amplified for unknown reasons. Secondary structures of viral RNA fragments were predicted using RNAfold software (http://rna.tbi.univie.ac.at//cgi-bin/RNAWebSuite/RNAfold.cgi). Short poly I:C (LMW poly I:C) (Cat# tlrl-picw) was purchased from InvivoGen.

### Western Blotting Analysis

Cells were washed with PBS and lysed with NP-40 lysis buffer [20 mM Tris-HCl (pH7.5), 125 mM NaCl, 10% glycerol, 1% NP-40, 1 mM EDTA, 30 mM NaF, and 5 mM Na_3_VO_4_] in the presence of a complete protease inhibitor cocktail (Roche). The cell lysates were incubated on ice for 30 min and were then centrifuged for 20 min at 15,000 rpm and at 4°C. The supernatants were transferred to 1.5 ml tubes and suspended in 2xLaemmil sample buffer containing β-mercaptoethanol. The cell lysates were boiled for 5 min at 95°C. All samples and a protein marker (Bio-Rad) were each loaded into separate wells for SDS-PAGE in a Tris-glycine-SDS buffer before being transferred onto PVDF membranes. The membranes were blocked with 5% skim milk (NACALAI TESQUE) in the rinse buffer [0.1% Tween 20, 10 mM Tris-HCl (pH7.5), 0.8% NaCl, and 1mM EDTA] and was incubated with 1st Ab (1:1,000) at 4°C overnight, and then, they were incubated with HRP-conjugated secondary Ab (1:10,000) for 60 min at room temperature. The immunoblots were visualized with ECL Prime Western Blotting Detection Reagent (GE Healthcare) and detected using a ChemiDoc Touch Imaging System (Bio-Rad). Anti-FLAG (Cat# F3165) and anti-HA (Cat# H6908) antibodies were purchased from Sigma Aldrich. Anti-Myc antibody was purchased from BioLegend (Cat# 626802). Anti-RIG-I antibody (Alme-1) was purchased form AdipoGen (Cat# AG-20B-009). Anti-MDA5 antibody (D74E4) was purchased form Cell Signaling Technology. Anti-Rabbit IgG HRP-linked whole Ab (Cat#NA934) and anti-Mouse IgG HRP-linked whole Ab (Cat# NA931) were purchased from (GE Healthcare). Anti-IRF-3 (D614C) and anti-phospho-IRF3 (Ser396) (4D4G) antibodies were purchased from Cell Signaling Technology.

### Immunoprecipitation

HEK293FT (5 x 10^5^) cells were cultured in a 6-well plate for 24 h and then transfected with expression vectors using Lipofectamine 2000 reagent. Total plasmid amounts were maintained at 1 μg by adding empty plasmids. The cells were harvested and washed with PBS 24 h after transfection and then lysed with a lysis buffer containing protease inhibitor cocktail. The cell lysates were kept on ice for 30 min and were centrifuged at 15,000 rpm and 4°C for 20 min. The supernatants were transferred into 1.5 ml tubes. The lysates were pretreated with protein G sepharose beads at 4°C for 60 min with rotation and then centrifuged to deplete the protein G sepharose beads. Anti-FLAG (1:150) Ab was added to the cell lysates, and then the lysate was incubated for 2 h at 4°C with rotation. Washed protein G sepharose beads were added to the lysates containing the Ab, and incubated overnight with rotation. Then, the protein G sepharose beads were collected by centrifugation and washed three times with the lysis buffer. The precipitated samples were analyzed by Western blotting.

### Reporter Gene Assay

HEK293 (1 x 10^5^) cells were seeded in 24-well plates in triplicate and transfected with an expression vector together with p-125 Luc reporter (100 ng/well), and phRL-TK (10 ng/well, Promega) plasmids. The phRL-TK (HSV-thymidine kinase promoter) plasmid encoded *Renilla* luciferase and was used as an internal control. An empty plasmid was added to ensure that each transfection received the same total DNA amount. Twenty-four hours after transfection, luciferase assays were performed using a Dual Luciferase Assay Kit (Progemga). Luciferase activity was normalized to that of *Renilla* luciferase.

### Quantitative Real-Time PCR

Total RNA was isolated form cells using TRIzol reagent (Invitrogen), according to the manufacture’s protocol. cDNA was generated from the total RNA using a High-Capacity cDNA Reverse Transcription Kit (Applied Biosystems). The target mRNA was quantified with Power SYBR Green Master Mix (Applied Biosystems) using the ViiA-7 Real-Time PCR system (Applied Biosystems). In each experiment, the expression of genes (Target/GAPDH) was determined using threshold cycle (Ct) values standardized to GAPDH (housekeeping gene), applying the 2^-(ΔΔCt)^ method, according to manufactures’ instruction and previous studies (31, 32). To compare the small difference in cytokine expression in **Figures 4F, G**, we calculated fold increase by dividing the expression of gene value at each time point by that at 0 hr time point in WT or Riplet KO cells.

### Quantification and Statistical Analysis

All qPCR assays and reporter gene assays were performed in triplicate (n = 3). Error bars represent standard deviation (SD). Statistical significance (p-value) was determined using a two-tailed Student’s *t*-test, one-way ANOVA, or two-way ANOVA using Prism v7.0a (GraphPad Software) and MS-Excel (Microsoft Corp) software. *p < 0.05.

## Result

### Cytoplasmic RNAs of SARS-CoV-2-Infected Cells Activated RIG-I and MDA5 in HEK293 Cells

**Figure 1:**
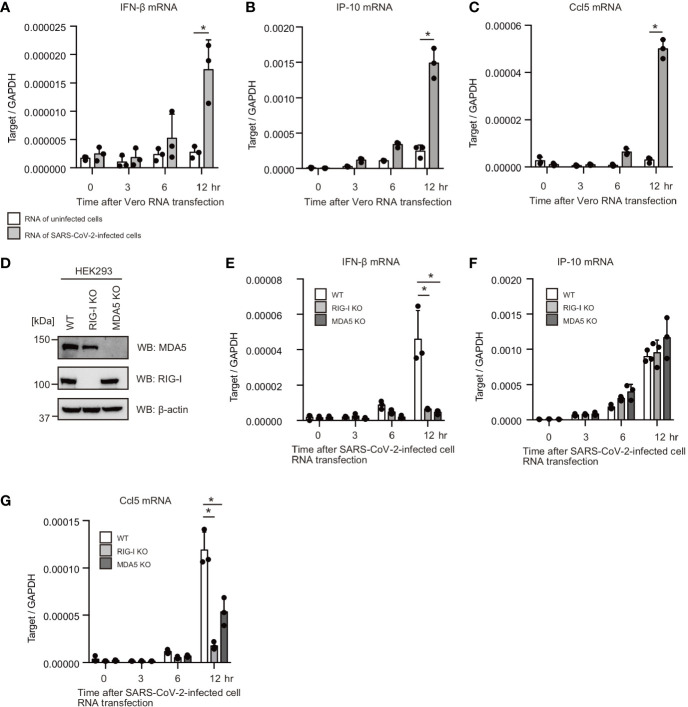
Total RNAs of SARS-CoV-2-infected cells induce type I IFN expression. **(A–C)** Total RNAs were extracted from VeroE6/TMPRSS2 cells infected with mock or SARS-CoV-2 for 24 h. 1 μg of extracted RNA was transfected into HEK293 cells, and IFN-β **(A)**, IP-10 **(B)**, and Ccl5 **(C)** mRNA expression at indicated time points were determined by RT-qPCR (n = 3). The data shown is representative of two independent experiments. **(D)** Whole cell extracts of WT, RIG-I KO, and MDA5 KO HEK293 cells were prepared and subjected to SDS-PAGE. The proteins were detected by western blotting using anti-RIG-I, MDA5, and β-actin antibodies. **(E–G)** 1 μg of the total RNA from VeroE6/TMPR22S infected with SARS-CoV-2 was transfected into WT, RIG-I KO, and MDA5 KO cells. IFN-β **(D)**, IP-10 **(E)**, and Ccl5 **(F)** mRNA expression at indicated time points were determined by RT-qPCR (n = 3, *p < 0.05, t-test). The data shown is representative of two independent experiments.

First, we investigated whether human cells can respond to cytoplasmic RNAs of SARS-CoV-2-infected cells. VeroE6/TMPRSS2 cells were infected with SARS-CoV-2 (JPN/TY/WK-521) for 1 d, and total RNA was extracted from SARS-CoV-2-infected and mock-infected cells. Then, total RNAs were transfected into HEK293 cells. Interestingly, total RNA of SARS-CoV-2-infected cells increased IFN-β, Ccl5, and IP-10 mRNA expression in HEK293 cells, but RNA from uninfected cells did not (**Figures 1A–C**). RIG-I and MDA5 are the primary cytoplasmic viral RNA sensors; thus, we generated RIG-I and MDA5 knockout HEK293 cells using the CRISPR-Cas9 system. We confirmed mutations on RIG-I and MDA5 by DNA sequencing, and the expression of the RIG-I and MDA5 proteins was barely detectable (**Figure 1D**). Then, we investigated the effects of RIG-I KO and MDA5 KO on cytokine expression. Interestingly, RIG-I KO markedly reduced IFN-β and Ccl5 mRNA expression (**Figures 1E–G**). MDA5 KO also reduced IFN-β and Ccl5 mRNA expression in response to RNA from SARS-CoV-2-infected cells (**Figures 1E–G**). These data suggest that the cytoplasmic viral RNA sensors recognize cytoplasmic RNAs of SARS-CoV-2 infected cells. Unlike IFN-β and Ccl5, IP-10 expression was not reduced by either RIG-I KO or MDA5 KO (**Figure 1F**), implying that either RIG-I or MDA5 would be sufficient to induce IP-10 expression.

### Viral RNA Regions Recognized by the Cytoplasmic Viral RNA Sensors

**Figure 2:**
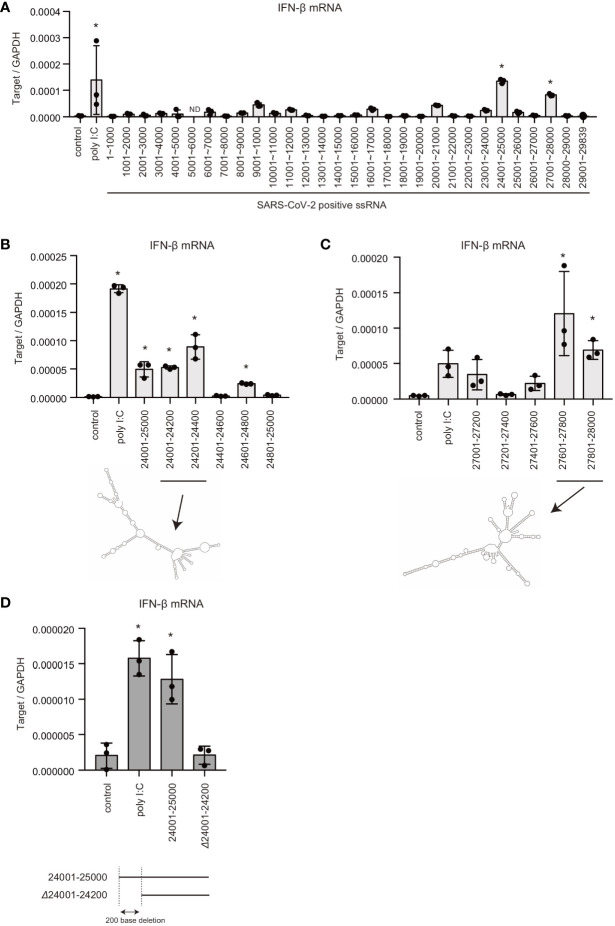
Viral genomic RNA regions that induce type I IFN expression. **(A)** SARS-CoV-2 viral RNA fragments were synthesized *in vitro* with T7 RNA polymerase. Each viral RNA fragment or short poly I:C was transfected into HEK293 cells for 6 h, and type I IFN expression was determined by RT-qPCR. The data represent the mean ± SD (n = 3, *p < 0.05, One-way ANOVA)). The data shown is representative of two independent experiments. **(B, C)** Short poly I:C or viral RNA fragments containing depicted viral RNA regions were transfected into HEK293 cells, and type I IFN expression was determined by RT-qPCR. The data represent the mean ± SD (n = 3, *p < 0.05, One-way ANOVA)). Predicted secondary structures of viral RNA 24001–24400 and 27601–28000 are shown in lower panel. The data shown is representative of two independent experiments. **(D)** Viral RNA fragments, 24001–25000 and 24200–25000 (Δ24001-24200), were transfected into HEK293 cells, and type I IFN expression was determined by RT-qPCR. The data represent the mean ± SD (n = 3, *p < 0.05, One-way ANOVA). The data shown is representative of at least two independent experiments.

Previous studies showed that there are pathogen-associated molecular pattern (PAMP) RNA motifs or structures within viral genomic RNAs, which are preferentially recognized by pattern recognition receptors (PRRs) (5, 6, 33). Thus, we next investigated SARS-CoV-2 viral RNA genome region containing PAMP motif or structure. The viral RNA genome was segmented into 30 fragments, approximately 1 kb each, and each fragment was synthesized *in vitro* with T7 RNA polymerase. Each RNA fragment was transfected into HEK293 cells, and type I IFN expression was measured using RT-qPCR. Interestingly, the two viral RNA fragments containing 24001–25000 or 27001–28000 significantly induced type I IFN expression (**Figure 2A**).

To further investigate the regions responsible for inducing type I IFN, the 24001–25000 fragment was further segmented into five fragments, approximately 200 bases each. Although, the fragments containing 24401–24600 or 24801–24500 did not induce IFN-β mRNA expression: other viral RNA genome fragments induced IFN-β mRNA expression (**Figure 2B**). Next, the 27001–28000 fragment was segmented into five fragments, and each fragment was transfected into HEK293 cells. The 27201–27400 region failed to induce type I IFN expression, whereas the other regions induced IFN-β mRNA expression (**Figure 2C**). There were predicted long stem regions within the 24001–24400 and 27601–28000 fragments (**Figures 2B, C**), which is consistent with the notion that RIG-I assembles along dsRNAs. Deletion of first 200 bases of the 24001–25000 fragment markedly reduced IFN-β mRNA expression (**Figure 2D**). These data suggest that the viral RNA region 24001–24200 is crucial for recognition by RLRs. We do not exclude the possibility that other regions are also recognized by PRRs.

**Figure 3:**
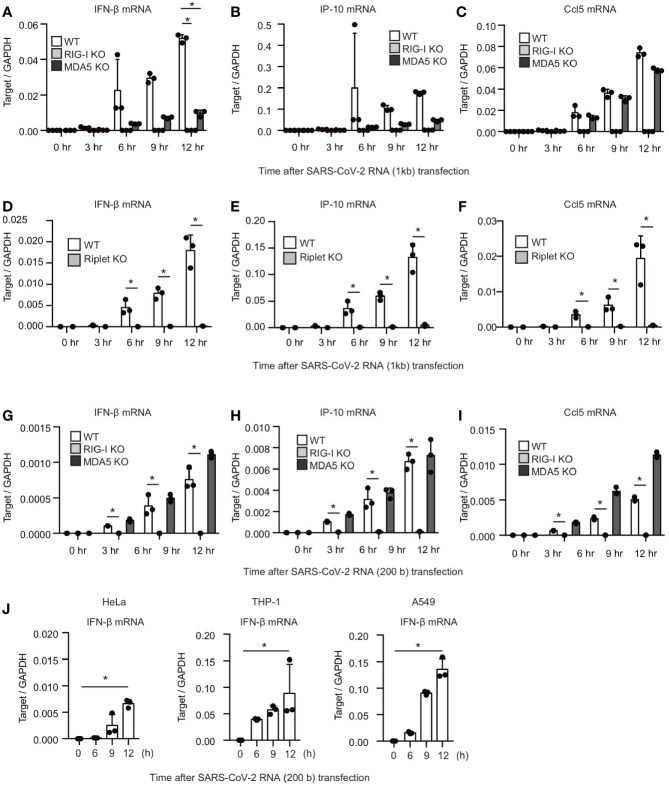
RIG-I and MDA5 recognizes viral RNA fragment of SARS-CoV-2. **(A–I)** The viral RNA fragment containing 24001–25000 **(A–F)** or 24001–24200 region **(G–I)** was transfected into WT, RIG-I KO, MDA5 KO, and Riplet KO HEK293 cells, and then the expression of the cytokines was determined by RT-qPCR. The data represent the mean ± SD (n = 3, *p < 0.05, t-test). The data shown is representative of at least two independent experiments. **(J)** The viral RNA fragment containing 24001–24200 region was transfected into HeLa, A549, and THP-1 cells, and then expression of IFN-β was determined by RT-qPCR. The data represent the mean ± SD (n = 3, *p < 0.05, t-test). The data shown in representative of at least two independent experiments.

Next, we investigated the effect of RIG-I KO and MDA5 KO on the type I IFN expression in response to viral RNA fragments. The 24001–25000 fragment was transfected into WT, RIG-I KO, and MDA5 KO cells. Interestingly, RIG-I KO and MDA5 KO markedly reduced IFN-β, IP-10, and Ccl5 mRNA expression (**Figures 3A–C**). In contrast, MDA5 KO failed to reduce the Ccl5 mRNA expression (**Figure 3C**), implying that the viral RNA-induced Ccl5 expression did not require MDA5 function. Riplet is an E3 ubiquitin ligase essential for RIG-I activation. Riplet KO also abolished IFN-β, IP-10, and Ccl5 mRNA expression as RIG-I KO did (**Figures 3D–F**).

We investigated the response to the 200-base viral fragment of the SARS-CoV-2 genome (24001–24200). MDA5 KO did not show any defect in cytokine expression in response to the short viral RNA fragment (**Figures 3G–I**), which is consistent with previous report showing that MDA5 barely respond to short dsRNA (2). In contrast, RIG-I KO markedly reduced cytokine expression in HEK293 cells (**Figures 3G–I**), suggesting that RIG-I is crucial for the response to the viral RNA fragment. Next, we investigated whether other types of cells can response to the viral RNA fragment. The viral RNA fragment could induce IFN-β expression in HeLa, A549, and THP-1 cells (**Figure 3J**). These data indicate that not only MDA5 but also RIG-I recognize SARS-CoV-2 viral RNAs in several human cells.

### Viral Proteins Suppress Host Protein Functions Required for Type I IFN Expression

**Figure 4:**
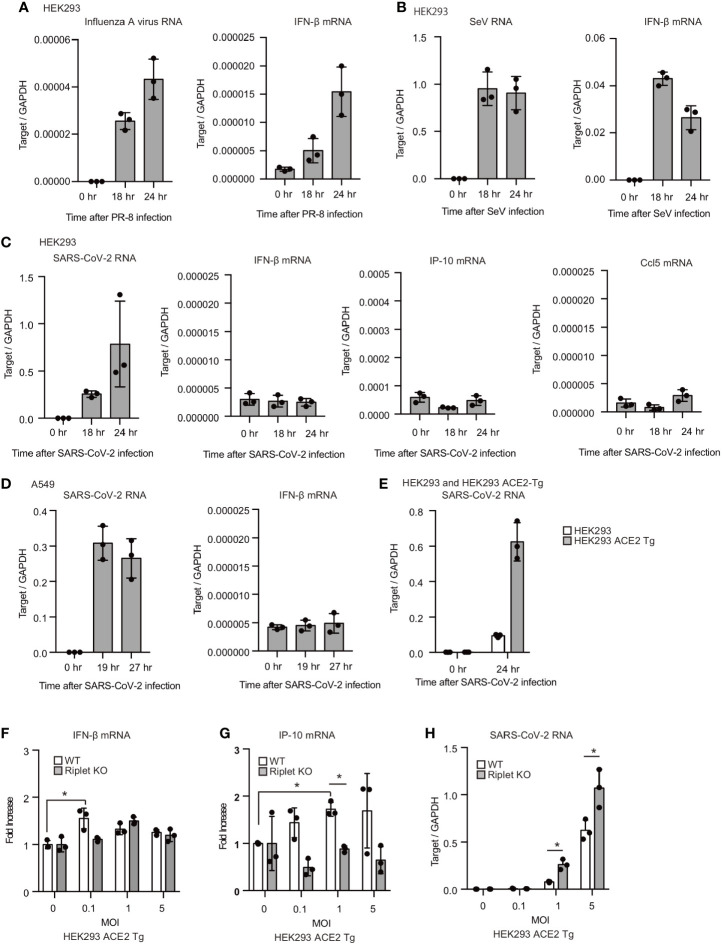
SARS-CoV-2 hardly induced type I IFN expression in human cells. **(A–C)** HEK293 cells were infected with influenza A virus, SeV, or SARS-CoV-2 at MOI = 1. Total RNA was extracted at the indicated time points, and RT-qPCR determined viral RNAs and IFN-β mRNA expression levels. The data represents the mean ± SD (n = 3). The data shown is representative of at least two independent experiments. **(D, E)** A549 **(D)** and human ACE2 overexpressing HEK293 cells (ACE2-Tg) **(E)** cells were infected with SARS-CoV-2 at MOI = 1. Total RNA was extracted at the indicated time points, and RT-qPCR determined viral RNAs and IFN-β mRNA expression levels. The data represent the mean ± SD (n = 3). The data shown is representative of at least two independent experiments. **(F–H)** WT and Riplet KO HEK293 ACE2 Tg cells were infected with SARS-CoV-2 at MOI = 0, 0.1, 1, and 5. Total RNA was extracted at 18 h after infection, and RT-qPCR determined IFN-β mRNA, IP-10 and SARS-CoV-RNA expression levels. The data are expressed as fold increase compared with expression level at 0 hr, and represent the mean ± SD (n = 3, *p < 0.05, t-test). The data shown is representative of at least two independent experiments.

We also investigated the cytokine expression after viral infection. For the controls, influenza A virus and Sendai virus (SeV) were used to compare with SARS-CoV-2. After influenza A virus or Sendai virus infection, viral RNA levels increased within the cells, and IFN-β mRNA expression was induced (**Figures 4A, B**). Previous studies showed that HEK293T cells are moderately susceptible to SARS-CoV-2 infection (34). We found that cytoplasmic viral RNA levels were increased in HEK293 cells after SARS-CoV-2 infection, suggesting replication of SARS-CoV-2 in HEK293 cells; however, IFN-β mRNA levels were not increased (**Figure 4C**). IP-10 and Ccl5 mRNA levels were also not increased by viral infection (**Figure 4C**). Next, we used a human lung epithelial cell line, A549. SARS-CoV-2 infection also failed to increase IFN-β mRNA levels in A549 cells (**Figure 4D**). Previous studies showed that ACE2 overexpression in human cells increases the susceptibility to SARS-CoV-2 (35, 36). As reported, the overexpression of ACE2 markedly increased the SARS-CoV-2 levels compared with WT HEK293 cells (**Figure 4E**). SARS-CoV-2 infection only marginally increased the cytokine expression in in ACE2-expressing HEK293 cells in our experimental condition (**Figures 4F, G**). Additionally, we found that Riplet KO moderately increased viral RNA levels in SARS-CoV-2 infected cells (**Figure 4H**). Considering weak type I IFN expression, these data suggest that SARS-CoV-2 could suppress host type I IFN response in HEK293 cells.

**Figure 5:**
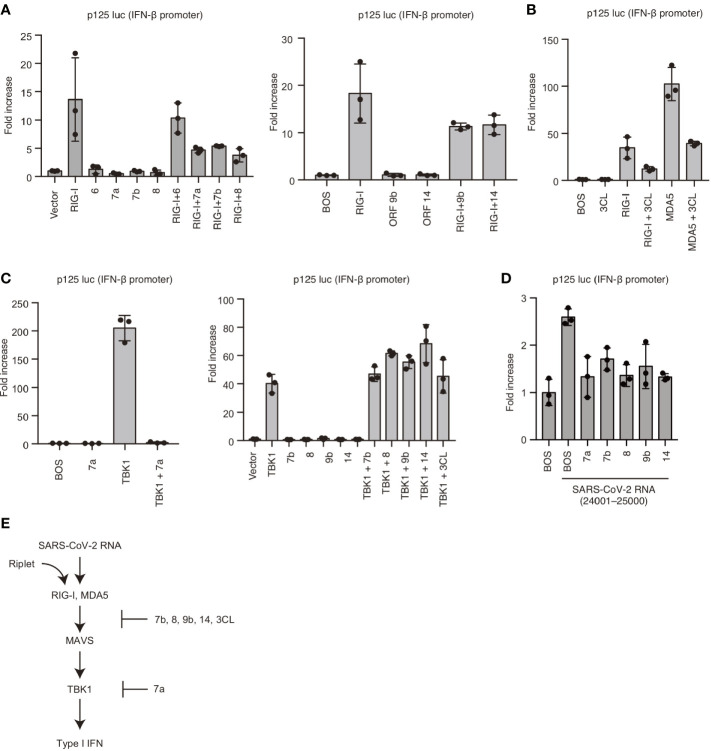
Viral proteins attenuated RIG-I-mediated signaling. **(A)** RIG-I, viral ORF 6, 7a, 7b, 8, 9b, and 14 expressing vectors were transfected into HEK293 cells with p125 luc plasmid for 24 h, and the luciferase activities were determined. The data represent the mean ± SD (n = 3). The data shown is representative of three independent experiments. **(B)** RIG-I, MDA5, and viral 3CL protein expressing vectors were transfected into HEK293 cells together with p125 luc plasmid for 24h, and the luciferase activities were determined. The data represent the mean ± SD (n = 3). The data shown is representative of three independent experiments. **(C)** TBK1, viral ORF 7a, 7b, 8, 9b, 14, and 3CL expressing vectors were transfected into HEK293 cells together with p125 luc plasmid for 24 h, and the luciferase activities were determined. The data represent the mean ± SD (n = 3). The data shown is representative of three independent experiments. **(D)** HEK293 cells were transfected with viral ORF 7a, 7b, 8, 9b, or 14 expressing vectors together with p125luc plasmid. Twenty-four hours after transfection, cells were then stimulated with the viral fragment trasnfection containing 24001–25000, then luciferase activities were determined. The data represent the mean ± SD (n = 3). The data shown is representative of three independent experiments. **(E)** Schematic representation of the cytoplasmic type I IFN expression pathway and viral protein-mediated suppression. The data shown is representative of three independent experiments.

To reveal the underlying mechanism, we cloned viral proteins and expressed them in human cells to investigate the effects of viral proteins on RIG-I and MDA5-mediated signaling. Interestingly, overexpression of viral 7a, 7b, 8, 9b, or 14 proteins reduced RIG-I-mediated IFN-β promoter activities (**Figure 5A**). Viral 3CL protein also reduced RIG-I and MDA5-mediated IFN-β promoter activities (**Figure 5B**). TBK1 is a protein kinase that plays a crucial role in RIG-I and MDA5-mediated type I IFN expression. Next, we investigated the effect of viral proteins on TBK1. Overexpression of TBK1 led to its auto-activation and induced IFN-β promoter activation (**Figure 5C**). Expression of Viral 7a could reduce TBK1-mediated IFN-β promoter activity, whereas viral 7b, 8, 9b, 14, and 3CL failed to reduce the activities (**Figure 5C**). We confirmed that viral 7a, 7b, 8, 9b, and 14 could reduce IFN-β promoter activity induced by SARS-CoV-2 viral RNA fragment (**Figure 5D**). These data suggest that viral 7a, 8, 9b, 14, and 3CL proteins can suppress RLR-mediated signaling; also, viral 7a targets TBK1 or its downstream factor (**Figure 5E**).

**Figure 6:**
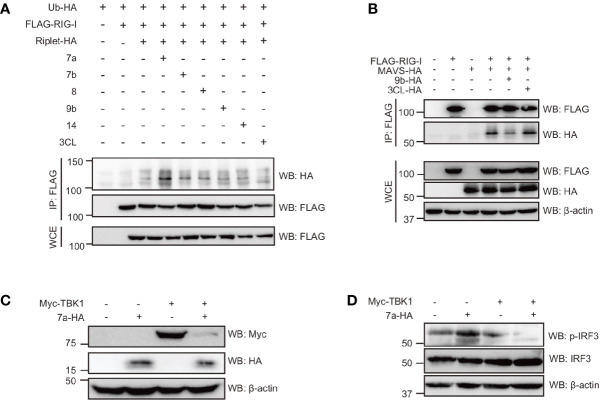
The viral protein targeted the components involved in RIG-I signaling. **(A)** HEK293FT cells were transfected with HA-tagged ubiquitin (HA-Ub), FLAG-tagged RIG-I, HA-tagged Riplet, viral ORF 7a, 7b, 8, 9b, 14, and 3CL expression vectors for 24 h as indicated, and then whole cell extract (WCE) was prepared. Immunoprecipitation was performed using an anti-FLAG antibody. The proteins were subjected to SDS-PAGE and detected by Western blotting with indicated antibodies. **(B)** HEK293FT cells were transfected with FLAG-tagged RIG-I, HA-tagged MAVS, viral 9b, and 3CL for 24 h, and then WCE was prepared. Immunoprecipitation was performed with anti-FLAG antibodies. The proteins were subjected to SDS-PAGE and detected by Western blotting. The data shown is representative of two independent experiments. **(C, D)** HEK293FT cells were transfected with myc-tagged TBK1 and viral 7a proteins for 24 h, and then WCE was prepared. The proteins were subjected to SDS-PAGE and detected by Western blotting as indicated antibodies. The data shown is representative of at least two independent experiments.

Since Riplet-mediated RIG-I ubiquitination was essential for RIG-I activation and type I IFN expression in response to SARS-CoV-2 RNAs, and we investigated whether viral proteins affect RIG-I ubiquitination. As previously reported, RIG-I polyubiquitination was increased by Riplet expression, but, viral 7a, 7b, 8, 9b, 14, and 3CL barely affected RIG-I polyubiquitination (**Figure 6A**). Next, we investigated the effect of viral proteins on RIG-I and MAVS interaction. MAVS was co-immunoprecipitated with RIG-I, and the amount of co-immunoprecipitated MAVS were reduced by viral 9b expression (**Figure 6B**). These data suggest that viral 9b inhibits RIG-I and MAVS interaction, and is consistent with the genetic data that 9b suppresses RIG-I-mediated signaling but not TBK1-mediated signaling. Interestingly, the viral 7a expression markedly reduced TBK1 protein levels (**Figure 6C**), consistent with our observation that 7a reduced TBK1-mediated signaling. TBK1 phosphorylates the IRF3 transcription factor essential for IFN-β mRNA expression. Interestingly, the viral 7a expression reduced phosphorylation of IRF-3 (**Figure 6D**). These data also support our conclusion that viral 7a protein destabilizes TBK1.

## Discussion

RLR are the primary cytoplasmic viral RNA sensors and elicit type I IFN responses after viral infection (1). This study revealed that both RIG-I and MDA5 recognize viral RNAs in the cytoplasm. We found that two viral RNA regions were preferentially recognized by RIG-I and MDA5, inducing type I IFN and other cytokine expressions. However, viral proteins can suppress RIG-I and MDA5-mediated type I IFN expression. Viral 9b inhibited the interaction between RIG-I and MAVS, and viral 7a destabilized TBK1 protein. These findings are consistent with recent findings showing that SARS-CoV-2 induces minor type I IFN expression than other pro-inflammatory cytokines.

MDA5 recognizes long dsRNA and makes a nucleoprotein filament along the dsRNA, whereas RIG-I binds shorter dsRNA and requires tri or diphosphate at the 5′ end (2, 37, 38). Thus, RIG-I and MDA5 play distinct roles in recognizing viral RNA. Indeed, RIG-I primarily recognizes influenza A virus and SeV, whereas MDA5 primarily recognizes EMCV and polioviruses (39–41). Both RIG-I and MDA5 recognizes some virus, such as Japanese encephalitis virus (JEV), Dengue virus, and West Nile virus (40, 42). Recent studies showed that MDA5 mainly recognizes SARS-CoV-2 in human lung epithelial cells (27, 34, 43). However, a study shows that RIG-I KO reduced the type I IFN expression in response to SARS-CoV-2 RNA (44). Considering that SARS-CoV-2 viral RNAs were detected in various tissues, we prefer the interpretation that both MDA5 and RIG-I influence SARS-CoV-2 recognition in some cell types.

Recently, Yamada T et al. reported that RIG-I bound to viral RNA of SARS-CoV-2 and suppress viral replication in an IFN-independent manner (45). Although RIG-I bound to viral RNAs, WT cells failed to induce type I IFN expression in response to SARS-CoV-2 infection (45), which is consistent with our observation that SARS-CoV-2 barely induced type I IFN response in HEK293 cells. Generally, viruses have evolved to suppress the host innate immune responses (46). For instance, the NS3-4A protease of HCV cleaves MAVS, resulting in attenuated type I IFN expression (47). The NS1 protein of influenza A virus inhibits TRIM25 and Riplet-mediated RIG-I activation (48, 49). Here, we found that several SARS-CoV-2 viral proteins attenuate the host′s innate immune response. Therefore, we speculate that RIG-I could sense viral RNA, but viral proteins strictly inhibit RIG-I-dependent type I IFN expression. It is still possible that the expression of RIG-I and/or MDA5 was insufficient in cells used in this study to induce the expression of cytokines after SARS-CoV-2 expression. But, this possibility is weakened by our data showing the expression of RIG-I and MDA5 in HEK293 cells (**Figure 1D**).

Viral 9b protein of SARS-CoV-2 inhibited RIG-I and MAVS interaction, and 7a destabilized the TBK1 protein. Recently, researchers reported that the 9b protein interrupts the K63-linked polyubiquitination of NEMO required for NF-κB activation (44). Thus, the expectation exists that 9b suppresses host′s innate immune response at multiple steps. In addition to 7a and 9b, other viral proteins attenuated the innate immune response. These findings elucidate the ability of SARS-CoV-2 to suppress the host innate immune response. Also, these molecular mechanisms are important for understanding the innate immune responses in patients with COVID-19.

## Data Availability Statement

The original contributions presented in the study are included in the article/**Supplementary Material**. Further inquiries can be directed to the corresponding author.

## Author Contributions

HO supervised research and wrote the manuscript. TK, TN, and GW performed the experiments. All authors contributed to the article and approved the submitted version.

## Funding

This work was supported in part by Grants-in-Aid from JSPS KAKENHI Grant Number 19H03480 and Japan Agency for Medical Research and Development (AMED).

## Conflict of Interest

The authors declare that the research was conducted in the absence of any commercial or financial relationships that could be construed as a potential conflict of interest.
