# Supplementary material for: RIG-I-Like Receptor-Mediated Recognition of Viral Genomic RNA of Severe Acute Respiratory Syndrome Coronavirus-2 and Viral Escape From the Host Innate Immune Responses
Source: Front Immunol. 2021 Jun 25;12:700926. doi: 10.3389/fimmu.2021.700926 (PMC8267574; doi:10.3389/fimmu.2021.700926)
Supplement: Supplementary file 1 [file Table_1.pdf]

Table 1. Primers for PCR or qPCR

Construct / Oligo	5'-3' Sequence
IFN-β qPCR primers	Fowerd: tgggaggattctgcattacc<br>Reverse: cagcatctgctggttgaa
IP-10 qPCR primers	Fowerd: tccacgtgttgagatcattgc<br>Reverse: ggccttcgattctggattcag
CXCL5 qPCR primers	Fowerd: gctgtcatcctcattgctactg<br>Reverse: agcacttgccactggtgtag
GAPDH qPCR primers	Fowerd: caatatgattccacccatgg<br>Reverse: aatgagccccagcttctcc
SARS-CoV-2 qPCR primers	Fowerd: gcctcttctcgttcctcatcac<br>Reverse: agcagcatcaccgccattg
PR-8 (Influenza Virus) qPCR primers	Fowerd: tgaactgagaagcaggtactgg<br>Reverse: gaatgctgccataacggttg
Sendai virus qPCR primers	Fowerd: gttgttgagcaccttcgatac<br>Reverse: ggcctagtacgaacactgag

nCoV-RNA synthesis primers

1-1000	Fowerd: ttatcgaaattaatacgactcactatagggattaaaggtttataccttcc<br>Reverse: agaacgttccgtgtaccaag
1001-2000	Fowerd: ttatcgaaattaatacgactcactataggggaaaagagctatgaattgca<br>Reverse: tcagtgaatactgtgaaatt
2001-3000	Fowerd: ttatcgaaattaatacgactcactataggggactcattgatgctatgatg<br>Reverse: ccagactcatcaaataagta
3001-4000	Fowerd: ttatcgaaattaatacgactcactatagggtgagtttaaattggcttcac<br>Reverse: ttcttccagagttgttgtaa
4001-5000	Fowerd: ttatcgaaattaatacgactcactatagggactaagttcctcacagaaaa<br>Reverse: ggttaatgttgtctactgtt
5001-6000	Fowerd: ttatcgaaattaatacgactcactatagggtccacacgcaagttgtggac<br>Reverse: attggttgctctgtgaaata
6001-7000	Fowerd: ttatcgaaattaatacgactcactatagggtgatcttgtaccaaaccaac<br>Reverse: gtagattaaagaacctaggc
7001-8000	Fowerd: ttatcgaaattaatacgactcactatagggtcaaccgctgctttaggtgt<br>Reverse: cagacactaatgcctgatct
8001-9000	Fowerd: ttatcgaaattaatacgactcactatagggatgttggtgatagtgcggaa<br>Reverse: gctgatgttgcaaagtcagt
9001-10000	Fowerd: ttatcgaaattaatacgactcactatagggttgtgttttggctgctgaat<br>Reverse: acctgagttactgaagtcat
10001-11000	Fowerd: ttatcgaaattaatacgactcactatagggtctgatgttctttaccaacc<br>Reverse: tacccttgattgttcttttc
11001-12000	Fowerd: ttatcgaaattaatacgactcactatagggcacaccactggttgttactc<br>Reverse: accattttttcaaaggcttc
12001-13000	Fowerd: ttatcgaaattaatacgactcactatagggttcactactttctgttttgc<br>Reverse: actaccaagtaccatacctc
13001-14000	Fowerd: ttatcgaaattaatacgactcactatagggttagctgccacagtacgtct<br>Reverse: tcgcatggcatcacagaatt
14001-15000	Fowerd: ttatcgaaattaatacgactcactatagggaatgctggtattgttggtgt<br>Reverse: tatatgcgaaaagtgcatct
15001-16000	Fowerd: ttatcgaaattaatacgactcactatagggcaaaacgtaatgtcatccct<br>Reverse: atagctaaagacacgaaccg
16001-17000	Fowerd: ttatcgaaattaatacgactcactatagggagatgcttacccacttacta<br>Reverse: attgctagaaaactcatctg
17001-18000	Fowerd: ttatcgaaattaatacgactcactataggggttgcaaattatcaaaaggt<br>Reverse: ttgccacattcctacgtgga

18001-19000	Fowerd: ttatcgaaattaatacgactcactatagggctttacaagctgaaaatgta<br>Reverse: tcgtgaagaactgggaattt
19001-20000	Fowerd: ttatcgaaattaatacgactcactatagggcattggtaaccctaaagcta<br>Reverse: ggcatttctaaataagtcta
20001-21000	Fowerd: ttatcgaaattaatacgactcactatagggcgtaatggtgttcttattac<br>Reverse: cccatttattagctgtatgt
21001-22000	Fowerd: ttatcgaaattaatacgactcactatagggatctcattattagtgatatg<br>Reverse: actcactttccatccaactt
22001-23000	Fowerd: ttatcgaaattaatacgactcactatagggtcagagtttattctagtgcg<br>Reverse: aagtaacaattaaaaccttc
23001-24000	Fowerd: ttatcgaaattaatacgactcactatagggtcctttacaatcatatggtt<br>Reverse: gaaaagtagatcttcaataa
24001-25000	Fowerd: ttatcgaaattaatacgactcactatagggaacaaagtgacacttgcaga<br>Reverse: taaaatatttatctaactcc
25001-26000	Fowerd: ttatcgaaattaatacgactcactatagggagaatcatacatcaccagat<br>Reverse: ctggtaatagtctgaagtga
26001-27000	Fowerd: ttatcgaaattaatacgactcactatagggctgtactcaactcaattgag<br>Reverse: acagtgatttctttaggcag
27001-28000	Fowerd: ttatcgaaattaatacgactcactatagggtgctacatcacgaacgcttt<br>Reverse: ataccatttagaatagaagt
28001-29000	Fowerd: ttatcgaaattaatacgactcactatagggattagagtaggagctagaaa<br>Reverse: tcagcagcagatttcttagt
29001-29839	Fowerd: ttatcgaaattaatacgactcactataggggggcttctaagaagcctcggc<br>Reverse: gtcattctcctaagaagcta
